# Prevalence of Tissue* BRCA* Gene Mutation in Ovarian, Fallopian Tube, and Primary Peritoneal Cancers: A Multi-Institutional Study

**DOI:** 10.31557/APJCP.2020.21.8.2381

**Published:** 2020-08

**Authors:** Arb-Aroon Lertkhachonsuk, Prapaporn Suprasert, Tarinee Manchana, Thannaporn Kittisiam, Nuttavut Kantathavorn, Tharintorn Chansoon, Surapan Khunamornpong, Natkrita Pohthipornthawat, Siriwan Tangjitgamol, Taksa Luasiripanthu, Chinachote Teerapakpinyo, Shanop Shuangshoti, Nareenart Iemwimangsa, Wasun Chantratita

**Affiliations:** 1 *Division of Gynecologic Oncology, Department of Obstetrics and Gynaecology, Faculty of Medicine, Ramathibody Hospital, Mahidol University Bangkok, Thailand. *; 2 *Division of Gynecologic Oncology, Department of Obstetrics and Gynecology, Faculty of Medicine, Chiang Mai University, Chiang Mai, Thailand. *; 3 *Division of Gynecologic Oncology, Department of Obstetrics and Gynecology, Faculty of Medicine, Chulalongkorn University and King Chulalongkorn Memorial Hospital, Bangkok, Thailand. *; 4 *Division of Gynecologic Oncology, Department of Obstetrics and Gynecology. Faculty of Medicine Vajira Hospital, Navamindradhiraj University, Bangkok, Thailand. *; 5 *Faculty of Medicine and Public Health, HRH Princess Chulabhorn College of Medical Science, Chulabhorn Royal Academy, Bangkok, Thailand. *; 6 *Chulabhorn Hospital, HRH Princess Chulabhorn College of Medical Science, Chulabhorn Royal Academy, Bangkok, Thailand. *; 7 *Department of Pathology, Faculty of Medicine, Ramathibodi Hospital, Mahidol University, Bangkok, Thailand. *; 8 *Department of Pathology, Faculty of Medicine, Chiang Mai University, Chiang Mai, Thailand. *; 9 *Division of Gynecologic Pathology and Cytology, Department of Obstetrics and Gynecology, Faculty of Medicine, Chulalongkorn University and King Chulalongkorn Memorial Hospital, Bangkok, Thailand. *; 10 *Department of Anatomical Pathology, Faculty of Medicine Vajira Hospital, Navamindradhiraj University, Bangkok, Thailand. *; 11 *Chulalongkorn GenePRO Center, Department of Pathology, Faculty of Medicine, Chulalongkorn University, Bangkok, Thailand. *; 12 *Department of Pathology, Faculty of Medicine, Chulalongkorn University, Bangkok, Thailand. *; 13 *Center for Medical Genomics, Faculty of Medicine Ramathibodi Hospital, Mahidol University, Bangkok, Thailand. *

**Keywords:** BRCA mutation, ovarian cancer, fallopian tube cancer, peritoneal cancer

## Abstract

**Background and objective:**

Ovarian, fallopian tube, or primary peritoneal cancer patients with *BRCA* gene mutation have enhanced sensitivity to platinum-based regimens and PARP inhibitors. However, the knowledge regarding *BRCA* mutation in Thai patients is limited. This study aimed at identifying the prevalence and characteristics of somatic and germline *BRCA 1* and *2* mutations in Thai patients with these cancers.

**Materials and Methods:**

The paraffin blocks of tumors with histology of high grade serous, high grade endometrioid, or clear cell carcinoma obtained between June 2016 and December 2017 were analyzedto evaluate *BRCA* mutation using next-generation sequencing system. Blood or normal tissue paraffin blocks of positive patients were further tested for germline *BRCA* mutation.

**Results:**

Tissue paraffin blocks of 178 patients were collected but only 139 were analyzed. Positive *BRCA* mutation was identified in 24 patients (17.3%): *BRCA*1 in 13 cases, *BRCA2* in 10 cases, and *BRCA1* and *2* in the rest one. Germline mutation study in blood or normal tissue in 23 positive patients revealed *BRCA* mutation in 14 cases, *BRCA1* in 8 cases and *BRCA 2 *in 6 cases. Overall, the prevalence of somatic and germline mutation was 6.5% (9 out of 138 patients) and 8.7% (14 out of 138 patients), respectively. The most common histology associated with *BRCA* mutation was high grade serous cancer (27.3%). No significant difference was found between patients with or without *BRCA* mutation in terms of stage, outcome, platinum status, and survival outcome.

**Conclusion:**

*BRCA* mutation was demonstrated in less than 10% of Thai ovarian cancer patients. Higher rate of mutation was found in high grade serous cancer.

## Introduction

Epithelial ovarian cancer (EOC) is the 6th most common cancer among Thai females with the age standardized rate of 6.0 per 100,000 females (Wilailak and Lertchaipattanakul, 2016; Torre et al., 2018). 

Among prognostic factors affecting survival, stage of the disease is the most important one. For instance, 2-year survival rate ranged from 90% in early and 40-50% in advanced stages (Webber and Friedlander, 2017). Another important prognostic factor is the response to first-line chemotherapy or platinum-sensitivity status. When the recurrences occur either later than 6 months or sooner after the last therapy, the diseases are regarded as platinum-sensitive or platinum-resistant, respectively. Platinum-based chemotherapy is usually used for platinum-sensitive disease; whereas, other non-platinum chemotherapies can be the option for platinum-resistant diseases (Jansaka and Suprasert, 2014; Webber and Friedlander, 2017). 

Unfortunately, the patients with platinum-sensitive disease, who have responded to platinum-based chemotherapy re-induction, may have subsequent recurrences. The second-relapse generally have lower responses to treatment with platinum or other chemotherapeutic drugs (Jansaka and Suprasert, 2014; Webber and Friedlander, 2017). Hence, maintenance therapy after primary treatment has emerged in order to extend the progression-free period and survival. 

Among drugs that are used as maintenance therapy, poly (adenosine diphosphate [ADP]) ribose polymerase (PARP) inhibitor (PARPi) is a recent efficient agent. PARP is an enzyme that repairs the single-strand break DNA in tumor cells. The mechanism of PARPi action is through inhibiting this enzyme, preventing DNA repair, and leading to further damage to the other DNA strand or double-strand break DNA. The double-strand break DNA can be repaired to normal via homologous recombinant pathway by* BRCA1/2* gene. In patients with *BRCA1/2 *mutation, the repair mechanism cannot take place leading to cell death eventually (Hennessy et al., 2010; Ledermann et al., 2014; Pujade-Lauraine et al.,2017). Evidence-based data from previous studies showed that PARPi could prolong remission in patients especially those with platinum-sensitive recurrent ovarian cancer. This benefit of PARPi maintenance therapy on progression-free survival was evidenced especially in patients with either germline or somatic *BRCA1/2* mutation (Hennessy et al., 2010; Cancer Genome Atlas Research Network 2011; Ledermann et al., 2014; Pujade-Lauraine et al., 2017). Hence, knowing the status of *BRCA1/2* mutation in ovarian cancer patients is important in order to support a decision a clinician for selecting an appropriate treatment option for the patients. 

The prevalence of germline *BRCA1/2* mutation, according to previous reports, ranged from 14 to 29% in Asia (Wu et al., 2017; Enomoto et al., 2019; Kwon et al., 2019). Higher percentages of mutation were found in some types of EOC, such as high grade serous cancer, which was found to have *BRCA1/2* mutation as high as 28.5% (Kwon et al., 2019). 

With the possible ethnic influence, the prevalence of *BRCA1/2* mutation may differ across different countries. The genetic test, including testing of *BRCA1/2* mutation, in ovarian cancer patients had not been a standard practice in our country. Hence, the prevalence of *BRCA1/2* gene mutation in our population remained unknown. The Thai Gynecologic Cancer Society is aware of the increasing role of PARPi in treatment of ovarian cancer, so this study was conducted among tertiary centers for cancer care in Thailand to determine the *BRCA1/2* mutation status in Thai patients with EOC.

## Materials and Methods

The study was approved by local ethic committees of each participating institution. Inclusion criteria were as follows: Thai patients who had EOC, fallopian tube cancer, and primary peritoneal adenocarcinoma (PPA), operation between June 1, 2016 and December 31, 2017, histopathology of high grade serous, high grade endometrioid, or clear cell adenocarcinoma, available paraffin blocks for tissue processing, and available clinical data. In any cases of mixed cell types, these aforementioned histopathologic components must be at least 10% for the pathological processing and examination. 

*Laboratory data*

To confirm tumor histopathology and grade, the representative slides containing tumor from each institution were submitted for central pathologic review. The central pathologic review was done by a gynecologic pathologist (S.K.). The paraffin blocks of tumors were then randomly submitted to one of the two qualified genomic laboratories: 1) Center for Medical Genomics, Faculty of Medicine, Ramathibodi Hospital and 2) Chula GenePRO Center, Faculty of Medicine, King Chulalongkorn Memorial Hospital. Tumor cells percentage was reviewed by the pathologists (S.S). A cross-validation of the whole process from DNA extraction through data interpretation between both laboratories was executed in 10 samples before proceeding with the *BRCA1* and* BRCA2* mutation testing in the remaining cases. which were equally distributed to both laboratories.

DNA extraction and quality assessment were performed using QIAamp DNA FFPE Tissue kit and GeneRead DNA QuantiMIZE kits according to manufacturer’s recommendation (Qiagen, Hilden, Germany). *BRCA1 *and *BRCA2* mutations were analyzed using GeneReader next-generation sequencing (NGS) system (Qiagen, Hilden, Germany). Quality of data sequencing was examined using unique molecular index (UMI) coverage, including 100X > 90% and 60X >95% of *BRCA1* and *BRCA2* coding regions. The samples that showed inadequate DNA quality or UMI coverage were discarded to avoid false positive and false negative results. 

The variant classification was preliminarily evaluated by Ingenuity Variant Analysis (Qiagen) and manually reviewed according to the American College of Medical Genetics and Genomics (ACMG)/ the Association for Molecular Pathology (AMP) guidelines (Richards et al., 2015; Li et al., 2017). The final result was made after discussion and mutual agreement between both laboratories in all specimens. The patients, who had tissue *BRCA1/2* mutation, were counselled for germline testing. For germline testing, 5 milliliters of blood were drawn from patients, who had agreed and signed inform consent, using bidirectional direct sequencing. For other patients, the genetic testing was performed using normal tissue taken from the paraffin blocks. 

The prevalence of tissue *BRCA* mutation was calculated by dividing the number of patients who had *BRCA* mutation from tumor tissue to total number of patients who had tumor tissue testing. On the other hand, the prevalence of germline or somatic *BRCA* mutation was calculated different. This number would be divided by the total number of patients who had tumor tissue testing but excluded those patients with tissue *BRCA* mutation whose peripheral blood or the paraffin blocks with normal tissue were unavailable. 

*Clinical Data*

Clinical data of the patients extracted from their medical records. These data included age, stage of cancer according to the International Federation of Gynecology and Obstetrics (FIGO 2014), histopathology, dates of treatments, outcomes, status of disease, and living status. The patients who developed progression during the course of chemotherapy or had recurrence within 6 months after the completion of chemotherapy were classified as platinum-refractory and platinum-resistant, respectively. or else were classified as platinum-sensitive. Progression-free survival (PFS) was calculated from the date on surgery or the date of start chemotherapy in patients who underwent neoadjuvant chemotherapy until the date of progression, recurrence, or last evaluation. Overall survival (OS) was obtained from the same date that using for calculated PFS to date of death or last follow-up in patients who were alive at the end of the study. 

*Sample Size Calculation*

A target minimum sample size was calculated according to the previous publication reporting 10% prevalence of *BRCA1/2 *mutation in EOC (Zhao et al., 2017). With respect to alpha error at < 0.05 added with 10% loss of data, 160 patients were needed to be included in the study. 

*Statistical Analysis*

Statistical analysis of the data was carried out using IBM SPSS statistics for Windows program (version 22; IBM Corporation, Armonk, NY, USA). Descriptive data were presented as number and percentages, mean with SD or median with range. Patients with BRAC 1/ 2 mutation or without BRAC 1/ 2 mutation were compared using Chi-square or Fisher’s Exact test as appropriate. Survival data were analyzed with Kaplan Meier and compared between groups with log-rank test. A p-value of < 0.05 was considered as statistical significance.

## Results

A total of 178 patients from 5 cancer institutions were included in this study. the instiutions were Faculty of Medicine Ramathibodi Hospital, Mahidol University (n=52), Chiang Mai University Hospital, Chiang Mai University (n=44), King Chulalongkorn Memorial Hospital, Chulalongkorn University (n=43), Faculty of Medicine and Vajira Hospital, Navamindradhiraj University (n=31) , and Chulabhorn Hospital, Chulabhorn Royal Academy (n=8). All hospitals were in Bangkok, except for Chiang Mai University Hospital which is in the northern part of Thailand.

The mean age of the patients was 56.9 ± 11.4 years. Ovarian cancer was the most common diagnosis among the included patients (86.5%), followed by fallopian tube cancer, (9.6%) and primary peritoneal cancer (3.9%). About 57.4% of the patients were in advanced stage of the disease. High grade serous cancer was the most common histopathology (56.7%). Approximately 11.2% of patients had neoadjuvant chemotherapy prior to surgery; whereas, almost all (96.1%) received adjuvant chemotherapy. The most common first-line chemotherapy was carboplatin plus paclitaxel (68.0%). Complete response after primary treatment was achieved in 118 patients (66.3%) and 55 patients (30.9%) had later developed recurrence. 

Regarding *BRCA* results, there were only 139 patients whose paraffin-blocks could be further processed for the test. The others were disapproved due to suboptimal quality of DNA and sequencing data, so were excluded from further investigation. Out of 139 patients, the tissue *BRCA* gene mutation was found in 24 cases (17.3%): *BRCA 1* mutation in 13 cases (9.5%), *BRCA 2* mutation in 10 (7.2%), and both* BRCA 1* and *2* mutation in 1 case (0.7%). The remaining 11 patients (7.9%) were reported to have variants of uncertain significance (VUS). 

Table 1 shows *BRCA* gene mutation according to the histology. Only high grade serous cancer and clear cell carcinoma showed positive *BRCA* gene mutation: high grade serous cancer in 21 from 77 patients (27.3%) and clear cell carcinoma in 3 out of 55 ones (5.5%). Among 24 patients with *BRCA* positive, all except one could be pursued to have germline *BRCA* gene mutation testing by using peripheral blood in 18 patients and normal tissue of myometrium from paraffin block in the other 5 patients. The results showed germline positive in 14 patients, 8 *BRCA 1* mutation and 6 *BRCA2* mutation; 13 of which were high grade serous cancer and only 1 was clear cell carcinoma. Thus, among 138 patients who had the data of both germline and somatic, the percentages of germline *BRCA* gene mutation was 8.7% (14 out of 138 patients), while the percentage of somatic *BRCA* gene mutation was 6.5% (9 in 138 cases). There were 4 *BRCA* 1 mutation, 4 *BRCA*2 mutation , and 1 *BRCA*1 and 2 mutation. The patients who had germline mutation was significantly younger than those with only somatic mutation (48.4**±**10.6 versus 61.6**±**12.9 years, P =0.014). Thus, at least the prevalence rates of germline and somatic *BRCA* mutation in our study were 8.7% and 6.5%, respectively. 

Table 2 summarizes findings on *BRCA* gene mutation identified by next generation sequencing. Three mutation patterns were identified, including 11 frameshifts, 9 nonsenses, and 2 missenses. Missense mutations were observed only in *BRCA2* genes, while frameshift and nonsense were found in both* BRCA1* and *BRCA2*. Five novel mutations were observed in 6 patients; whereas, the others were known mutations reported in the NCBI database of genetic variation (dbSNP).

**Figure 1:**
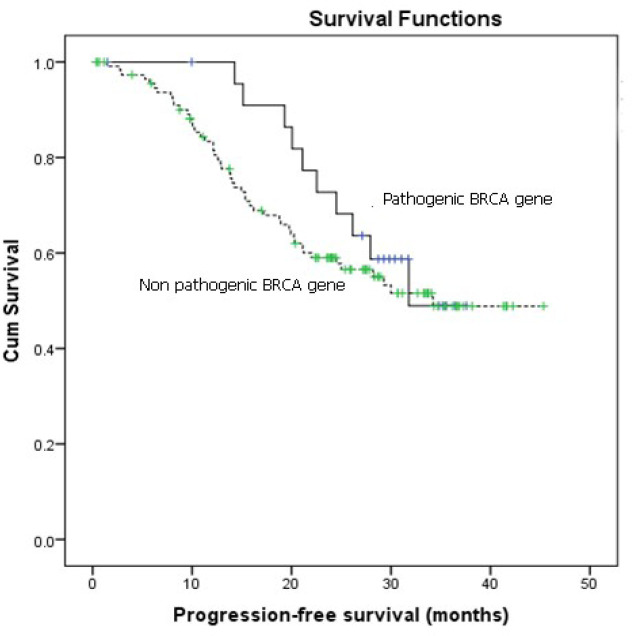
Progression –Free Survival in Patients with and without Pathogenic *BRCA* Gene. Median progression free survival: Pathogenic BRCA gene = 31.8 months vs. non-pathogenic BRCA gene = 30 months; 2 year progression free survival : Pathogenic BRCA gene = 68.2% (95%CI 48.8-87.6% ) vs. non- Pathogenic BRCA gene = 59% (95% CI 49.6-68.4); Log rank test P = 0.43; CI = confidence interval

**Figure 2:**
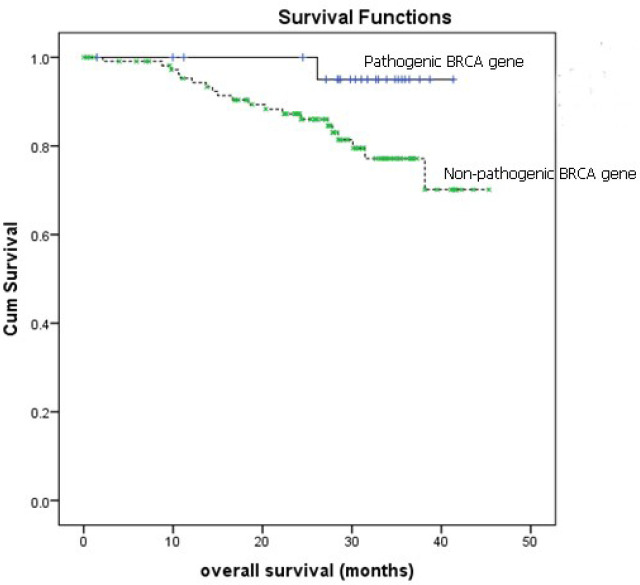
Overall Survival (OS) in Patients with and without Pathogenic *BRCA* Gene. Log rank test P = 0.077 2-yrs overall survival : non-pathogenic BRCA gene = 87.2%(95% CI 88.8-100%), pathogenic BRCA gene = 95% (95% CI 77.6-96.8%); CI = confidence interval

This study could not demonstrate any differences between patients with and without tissue *BRCA* gene mutation regarding age, type of cancer, stage, outcome after primary treatment, and platinum-sensitivity status (Table 3). Figure 1 and Figure 2 also revealed no differences between two groups of patients in terms of 2-year PFS and 2-year OS. However, the OS in patients with pathogenic *BRCA* gene tended to be better with 2-year OS of 95.0% (95% confidence interval 77.6-96.8%) compared to 87.2% (95% confidence interval 88.8-100%) in patients without pathogenic *BRCA* gene ( P = 0.077). 

**Table 1:** Clinico-Pathologic Data of the Patietns and Tumors (N=178)

Clinico-pathological features	N (%)
Diagnosis	
CA Ovary	154 (86.5)
CA tube	17 (9.6)
PPA	7 (3.9)
Histology	
Serous	101 (56.7)
Clear cell	69 (38.8)
Endometrioid	4 (2.2)
Mixed	4 (2.2)
Stage	
I	52 (29.2)
II	24 (13.5)
III	80 (44.9)
IV	22 (12.4)
Neoadjuvant chemotherapy	20 (11.2)
Adjuvant chemotherapy	171 (96.1)
Regimen	
Carboplatin + paclitaxel	121 (68.0)
Carboplatin	12 (6.7)
Carboplatin + Pegylated liposomal doxorubicin	37 (20.8)
Carboplatin + paclitaxel+Bevacizumab	1 (0.6)
Outcome after primary chemotherapy treatment Responses	
Complete response	118 (66.3)
Partial response	20 (11.2)
Stable disease	4 (2.2)
Progression	26 (14.6)
Missing data	10 (5.6)
Recurrence	55 (30.9)
Progression after response to chemotherapy	23 (12.9)
Platinum status	
Sensitive	129 (72.5)
Refractory	18 (10.1)
Resistant	21 (11.8)
Data not available (loss to follow-up)	10 (5.6)
Current status	
Alive with disease	55 (30.9)
Alive without disease	88 (49.4)
Death	27 (15.2)
Data not available (loss to follow-up)	8 (4.5)

**Table 2:** *BRCA* Gene Mutation According to the Histology (N=139)

Histology	BRCA result (%)						
	Positive				Negative	VUS	Total
	*BRCA1*	*BRCA2*	*BRCA1&2*	Total			
Serous	13 (16.9)	7 (9.1)	1 (1.3)	21 (27.3)	49 (63.6)	7 (9.1)	77
Clear	-	3 (5.5)	-	3 (5.5)	48 (87.3)	4 (7.3)	55
Endometrioid	-	-	-	-	4 (100)	-	4
Mixed	-	-	-	-	3 (100)	-	3
Total	13 (9.4)	10 (7.2)	1 (0.7)	24 (17.3)	104 (74.8)	11 (7.9)	139

VUS,Variant of Uncertain significance; Mixed histology included endometrioid caecinoma + clear cell carcinoma (2 cases), high grade serous carcinoma + celar cell carcinoma (1 case)

**Table 3:** Detail of *BRCA* Mutation in 24 Patients

No.	Gene	Mutation				Histology	Age of diagnosis	Germline / Somatic
		Nucleotide change	Amino acid change	Type of mutation	dbSNP		(years)	
1	*BRCA1*	c.624_625insAGGGATGAAATCAGGAACCA	p.(Pro209fsX)	Frameshift	rs397509302	Serous	53	Germline
2	*BRCA1*	c.697delG	p.(Val233Ter)	Nonsense	rs1555593298	Serous	64	Somatic
3	*BRCA1*	c.1265_1266dupAT	p.(Ser423IlefsX)	Frameshift	rs397508850	Serous	23	Germline
4**	*BRCA1*	c.1544_1550delAGGATTT	p.(Glu515fsX15)	Frameshift	-	Serous	31, 57	Germline, Germline
5	*BRCA1*	c.1789G>T	p.(Glu597Ter)	Nonsense	rs55650082	Serous	48	Germline
6**	*BRCA1*	c.1961delA	p.(Lys654SerfsX)	Frameshift	rs80357522	Serous	61	Somatic
7	*BRCA1*	c.2462dupA	p.(Asp821GlufsX2)	Frameshift	-	Serous	78	Somatic
8	*BRCA1*	c.2719G>T	p.(Glu907Ter)	Nonsense	rs876658593	Serous	47	Germline
9**	*BRCA1*	c.3037G>T	p.(Glu1013Ter)	Nonsense	-	Serous	58	Germline
10	*BRCA1*	c.3181delA	p.(Ile1061Ter)	Nonsense	rs80357702	Serous	54, 56	N/A***, Germline
11*,**	*BRCA1*	c.3769G>T	p.(Glu1257Ter)	Nonsense	-	Serous	58	Somatic
12**	*BRCA1*	c.4905_4914delGAAGCCAGAA	p.(Glu1635AspfsX)	Frameshift	-	Serous	45	Somatic
13*	*BRCA2*	c.145G>T	p.(Glu49Ter)	Nonsense	rs80358435	Serous	58	Somatic
14	*BRCA2*	c.755_758delACAG	p.(Asp252ValfsX24)	Frameshift	rs80359659	Serous	51	Germline
15	*BRCA2*	c.1321_1324delACTT	p.(Thr441GlnfsX18)	Frameshift	rs1064793572	Serous	64	Germline
16	*BRCA2*	c.1399_1402delAAGA	p.(Lys467GlufsX17)	Frameshift	rs398122726	Clear cell	46	Germline
17	*BRCA2*	c.1813delA	p.(Ile605TyrfsX9)	Frameshift	rs80359306	Clear cell	56	Somatic
18	*BRCA2*	c.4222C>T	p.(Gln1408Ter)	Nonsense	rs80358663	Serous	69	Somatic
19	*BRCA2*	c.4593dupA	p.(Val1532SerfsX2)	Frameshift	rs397507731	Clear cell	43	Somatic
20	*BRCA2*	c.7090G>T	p.(Glu2364Ter)	Nonsense	rs80358940	Serous	80	Somatic
21	*BRCA2*	c.8009C>T	p.(Ser2670Leu)	Missense	rs80359035	Serous	50	Germline
22	*BRCA2*	c.9154C>T	p.(Arg3052Trp)	Missense	rs45580035	Serous	45, 48	Germline, Germline

Mutations reported conform to HGVS guidelines on mutation nomenclature and are named according to the reference sequencing. NM_007294.3/LRG_292t1 and NM_000059.3/293t1 for *BRCA1* and *BRCA2, *respectively; Serous; high grade serous cystadenocarcinoma; *, mutations in the same patient. N/A; not tested; **, novel mutations; ***, not tested due to patient death and no adequate specimen.

**Table 4:** Clinical Data of the Patients According to *BRCA *Results (N=139)

	*BRCA*		*P*-value
	Pathogenic (N=24)	Non-pathogenic (N=115)	
Mean age	54.0	56.9	0.27*
Diagnosis			0.69#
Ovary	21 (87.5)	103 (89.6)	
Fallopian tube	3 (12.5)	9 (7.8)	
PPA		3 (2.6)	
Stage			0.07#
I	2 (8.3)	37 (32.2)	
II	5 (20.8)	15 (13.0)	
III	12 (50.0)	48 (41.7)	
IV	5 (20.8)	15 (13.0)	
Outcome after primary treeatment			0.88#
Complete response	18 (75.0)	77 (67.0)	
Partial response	2 (8.3)	14 (12.2)	
Stable disease	0	3 (2.6)	
Progression	2 (8.3)	15 (13.0)	
Data not available	2 (8.3)	6 (5.2)	
Platinum-sensitivity status			0.28#
Sensitive	21 (87.5)	85 (73.9)	
Refractory	0	8 (7.0)	
Resistant	1 (3.3)	16 (13.9)	
Data not available	2 (8.3)	6 (5.2)	

Non-pathogenic, *BRCA* negative + VUS (VUS = Variant of Uncertain significance); #, Fisher Exact test; *, Chi-square test

## Discussion

The present study found tissue *BRCA* mutation in high grade EOC, fallopian tube cancer, and primary peritoneal adenocarcinoma in 24 out of 139 patients (17.3%). Out of 24 patients, 14 patients revealed germline *BRCA 1/2* mutations. Thus, the prevalence of germline and somatic* BRCA* mutation in our study was at least 10.1% (14 in 138 cases) and 6.5% (9 in 138 cases), respectively. These rates corresponded to those reported by a Taiwanese’s study. Chao et al., (2016) investigated the occurrence of *BRCA 1/2* mutation in 99 formalin-fixed paraffin-embedded tumor samples of tumor and normal tissue obtained from Taiwanese ovarian cancer patients by using next-generation sequencing. They found *BRCA 1* in 7 cases and *BRCA 2* in 6 cases, reporting 12.1% prevalence. In their study, 4 cases (4%) had somatic mutation. Another study was done in Thailand by Manchana et al., (2019). They investigated the frequency of *BRCA* mutation in 87 Thai high grade serous cancer and high grade endometrioid ovarian cancer, including the fallopian and peritoneal cancers. They used peripheral blood DNA samplers, DNA extracted from formalin-fixed paraffin embedded blocks (FFPE), or a fresh tumor specimen to analyze *BRCA* mutation via the next generation sequencing system. The results showed germline *BRCA* mutation detection in 19 patients (21.8%), of which 14 (16.1%) were *BRCA1* mutation and 5 (5.7%) were *BRCA2* mutation. All positive patients revealed histology as high grade serous cancer. The difference in detection rates of *BRCA* mutation between our study and Manchanaet al., (2019)’s study was probably due to usage of differed specimens. Our study used only FFPE that might decrease the detection rate due to the variability of tumor samples and intra-tumoral heterogeneity which probably compromised the representativeness of the sample (da Cunha Colombo Bonadio, 2018); whereas, Manchana et al., (2019) used fresh tumor specimen or blood samples aside from FFPE to analyze *BRCA* prevalence.

The most frequent histology in patients with germline *BRCA* mutation was high grade serous carcinoma in a range of 5.5-16.7%, followed by endometrioid carcinoma (4.3-13%) ,and clear cell carcinoma (6.3-9.1%) (Vergote et al.,2016; da Cunha Colombo Bonadio, 2018) . However, in the present study, tissue *BRCA* mutation was found in 27.3% of high grade serous carcinoma and 5.5% of clear cell carcinoma, while none in endometrioid carcinoma. This non-similar outcome might be due to very small number of patients with high grade endometrioid carcinoma in our study. However,* BRCA2* mutation was found only in clear cell carcinoma. This finding corresponded to the previous reports suggested that *BRCA2 *mutation is common in this cell type (Goodheart et al., 2009; Manchana et al., 2019). However, 2 out of 3 patients with clear cell carcinoma had somatic *BRCA2* mutation (87.7%) and only 1 patient (33.3%) had germline *BRCA2* mutation.

Due to different reports on prevalence of *BRCA* mutation among various histologies, recently the American Society of Clinical Oncology (ASCO) suggest assessing either germline or somatic *BRCA* gene mutation in all non-mucinous ovarian, fallopian tube, and peritoneal cancers of high grade (Konstantinopoulos et al., 2020). Improvement of treatment outcome is possible through PARPi (Ledermann et al., 2014; Pujade-Lauraine et al., 2017; da Cunha Colombo Bonadio et al., 2018; Hennessy et al., 2010; Dougherty et al., 2017). Gori et al., (2019) summarized an initial step of *BRCA* mutation gene investigation by studying *BRCA 1/2* mutation on tumor tissue. The test for identifying spectrum of genomic rearrangements (i.e., deletions or duplications of one or more exons, or of the whole gene) yielded high sensitivity. In the patients, whose tissues were positive for* BRCA 1/2* mutation, genetic counseling and further investigation for germline mutation from their peripheral blood should be deferred as the next step of investigation. If the blood test be positive, these patients will benefit from PARPi therapy and should be under close surveillance for a possible development of second malignancy.

The major problem with using tissue sample for testing pathogenic gene was the quality of FFPE clinical specimens. Although next generation sequencing used in this study can sequence entire *BRCA 1/2 *coding regions, epigenetic and structural changes could not be detected in this study. Thirty nine DNA samples (21.9%) were unable to obtain *BRCA* mutation status due to insufficient DNA quality. Various factors affect FFPE DNA quality in molecular genomics test, such as tissue fixation time, storage time, and archive temperature (Einaga et al., 2017). In this study, we selected FFPE tissues blocks during June 2016 to December 2017 and finished the DNA extraction within 2018 (<3 years) to avoid long-term storage effect. Therefore, fixation time and archive temperature could lead to low DNA quality since the samples were collected from 5 institutions and there might be deviation of FFPE tissue handling and collection.

Regarding clinical characteristics between the patients with and without *BRCA* gene mutation, the present study did not find any significant differences in terms of age, disease, stage, primary outcome, platinum-sensitivity status, and survivals after treatment. Kuberlac et al., (2019) reported that their patients with both germline and somatic *BRCA* mutation had younger age and had significantly higher rate of complete pathologic response during the first and second platinum-sensitive relapse, and longer overall survival for advanced stage treated with neo-adjuvant chemotherapy when compared to 52 patients without *BRCA* mutation. Although this study also found younger age among *BRCA* mutation, the positive *BRCA* gene did not serve as a favorable prognostic factor. The small number of patients in this study may limit any meaningful clinical summary regarding the prognostic role of *BRCA* mutation. 

In conclusion, tissue *BRCA 1/2* mutation positive was presented in about 17% of high grade epithelial ovarian, fallopian, and peritoneal cancers. The most frequent histology was the serous cancer. 
